# Digital gait markers to potentially distinguish fragile X-associated tremor/ataxia syndrome, Parkinson’s disease, and essential tremor

**DOI:** 10.3389/fneur.2023.1308698

**Published:** 2023-12-07

**Authors:** Erin E. Robertson-Dick, Emily C. Timm, Gian Pal, Bichun Ouyang, Yuanqing Liu, Elizabeth Berry-Kravis, Deborah A. Hall, Joan A. O’Keefe

**Affiliations:** ^1^Department of Anatomy and Cell Biology, Rush University Medical Center, Chicago, IL, United States; ^2^Department of Neurological Sciences, Rush University Medical Center, Chicago, IL, United States; ^3^Department of Pediatrics, Rush University Medical Center, Chicago, IL, United States; ^4^Department of Biochemistry, Rush University Medical Center, Chicago, IL, United States

**Keywords:** fragile X-associated tremor/ataxia syndrome, Parkinson’s disease, essential tremor, gait, dual-task cognitive motor paradigms

## Abstract

**Background:**

Fragile X-associated tremor/ataxia syndrome (FXTAS), a neurodegenerative disease that affects carriers of a 55-200 CGG repeat expansion in the *fragile X messenger ribonucleoprotein 1* (*FMR1*) gene, may be given an incorrect initial diagnosis of Parkinson’s disease (PD) or essential tremor (ET) due to overlapping motor symptoms. It is critical to characterize distinct phenotypes in FXTAS compared to PD and ET to improve diagnostic accuracy. Fast as possible (FP) speed and dual-task (DT) paradigms have the potential to distinguish differences in gait performance between the three movement disorders. Therefore, we sought to compare FXTAS, PD, and ET patients using quantitative measures of functional mobility and gait under self-selected (SS) speed, FP, and DT conditions.

**Methods:**

Participants with FXTAS (*n* = 22), PD (*n* = 23), ET (*n* = 20), and controls (*n* = 20) underwent gait testing with an inertial sensor system (APDM^™^). An instrumented Timed Up and Go test (i-TUG) was used to measure movement transitions, and a 2-min walk test (2MWT) was used to measure gait and turn variables under SS, FP, and DT conditions, and dual-task costs (DTC) were calculated. ANOVA and multinomial logistic regression analyses were performed.

**Results:**

PD participants had reduced stride lengths compared to FXTAS and ET participants under SS and DT conditions, longer turn duration than ET participants during the FP task, and less arm symmetry than ET participants in SS gait. They also had greater DTC for stride length and velocity compared to FXTAS participants. On the i-TUG, PD participants had reduced sit-to-stand peak velocity compared to FXTAS and ET participants. Stride length and arm symmetry index during the DT 2MWT was able to distinguish FXTAS and ET from PD, such that participants with shorter stride lengths were more likely to have a diagnosis of PD and those with greater arm asymmetry were more likely to be diagnosed with PD. No gait or i-TUG parameters distinguished FXTAS from ET participants in the regression model.

**Conclusion:**

This is the first quantitative study demonstrating distinct gait and functional mobility profiles in FXTAS, PD, and ET which may assist in more accurate and timely diagnosis.

## Introduction

Fragile X-associated tremor/ataxia syndrome (FXTAS) is a progressive neurodegenerative disease that affects carriers of a ‘premutation’ size (55–200) CGG repeat expansion in the *fragile X messenger ribonucleoprotein 1* (*FMR1*) gene (1). Although the characteristic motor features are intention tremor and cerebellar gait ataxia, there is high phenotypic variability with some carriers also demonstrating parkinsonism, neuropathy, psychiatric symptoms, and/or executive function deficits and dementia (1–5). Because FXTAS was first described relatively recently (1) and has high phenotypic variability and overlap of symptoms with other more well-known movement disorders, patients are frequently given an incorrect initial diagnosis (6). This is especially the case when patients are seen by a primary care physician or general neurologist, or at a non-Fragile X clinic where FXTAS may not be readily recognized. At onset, FXTAS is most commonly diagnosed as Parkinson’s disease (PD) or essential tremor (ET), due to overlapping motor symptom profiles and lack of physician awareness of the disorder (6). Inaccurate diagnosis delays the initiation of targeted treatments and the provision of genetic counseling, negatively impacting health outcomes for patients and their families. Distinguishing the FXTAS disease profile, in terms of gait and functional mobility, from those of PD and ET may be critical in assisting with the differential diagnosis. We previously reported that tremorography using an inertial sensor system was able to distinguish between these three movement disorders, where higher kinetic tremor was found in FXTAS compared to PD patients and more bradykinesia was found in FXTAS compared to ET patients (7). Thus, quantitative measures of the prominent motor features of FXTAS, namely kinetic tremor and cerebellar gait ataxia, captured via wearable sensor technologies are likely to be beneficial in assisting clinicians with diagnostic accuracy.

Gait impairments are a common feature in FXTAS that can lead to significant disability. Our group first characterized the gait deficits in a small cohort of FXTAS participants during self-selected (SS) speed walking using an instrumented Timed Up and Go test (i-TUG) and found deficits in gait speed, rhythm, cycle phase, and variability as well as movement transitions compared to healthy controls (8). PD participants have shown similar gait deficits during SS walking using the i-TUG (9–15) and GAITRite^®^ walkway (16), with the addition of abnormalities in the domain of gait asymmetry in arm swing range of motion and stride length (9). Reduced stride velocity and cadence and increased double support time and gait asymmetry have been found in ET participants compared to controls during standard walking on the GAITRite^®^ walkway (17–19). Our group recently used an instrumented 2MWT under SS, fast as possible (FP) speeds, and with the addition of a cognitive dual task (DT) in FXTAS and found reduced stride length and velocity, swing time, and peak turn velocity and greater double limb support time and number of steps to turn as compared to controls under all three conditions. During the FP condition, stride length variability was increased, and cadence was reduced in FXTAS participants. Additionally, stride velocity variability under FP gait was significantly associated with the number of self-reported falls in the last year (20). Studies investigating FP walking in PD report reduced stride length and stride velocity and increased double support time compared to controls (21–23). No studies to date have examined gait under fast speed walking conditions in ET. DT cognitive and motor paradigms have been used previously to explore the interplay between cognition and gait in PD (24), ET (18), and FXTAS (20). PD participants have shown decreased gait velocity, stride length and swing phase time, and increased gait variability during DT gait testing (25–27), and similar interference effects have been seen in ET (28). We previously found greater dual task costs (DTC) of a verbal fluency task on peak turn velocity in men with FXTAS compared to women with FXTAS and controls (20). However, the gait profiles of FXTAS, PD, and ET patients have never been directly compared. This information is critical to inform clinicians of the distinct phenotypes in FXTAS compared to PD and ET, and aid in accurate diagnosis. Therefore, the objective of this study was to compare the gait profiles in FXTAS, PD, and ET using quantitative measures of gait during SS and FP speeds, and a DT cognitive-motor condition to determine whether these measures may be sensitive for distinguishing FXTAS from PD and ET.

## Methods

### Participants

FXTAS, PD, and ET participants were recruited through the Parkinson Disease and Movement Disorders Clinic at Rush University Medical Center (RUMC). Inclusion criteria for participants with movement disorders were: (1) A diagnosis of only one of these disorders made by a movement disorders neurologist at RUMC, (2) a *FMR1* gene test showing one allele with 55–200 CGG repeats for FXTAS participants and < 55 repeats on both alleles for PD and ET participants, (3) symptom onset at ≥ age 50, (4) mild to severe tremor, and (5) mild to moderate parkinsonism for PD participants with Hoehn & Yahr staging of PD score ≤ 3 (29). Exclusion criteria were: (1) A prior history of stroke with focal neurological deficit or any other neurological or muscular disease, (2) seizure disorder or past head trauma resulting in structural brain damage, (3) deep brain stimulation surgery, (4) presence of dyskinesia on neurological exam, and (5) clinical diagnosis of dementia as determined by the neurologist and/or neuropsychologist. Twenty healthy control subjects were recruited from RUMC or from the community. Inclusion criteria were: (1) a normal neurological examination, and (2) a *FMR1* gene test showing both alleles with <55 CGG repeats. Exclusion criteria were the same as for the FXTAS, PD, and ET participants, but also included a significant history of tremor, balance problems, falls, or dizziness. All participants were required to be between 50 and 90 years of age; this range was chosen because FXTAS typically develops after age 50. This study was approved by the RUMC Institutional Review Board, and all participants gave written informed consent.

### Gait assessments

Quantitative gait analysis was performed during a 25-meter instrumented 2-min walk test (2MWT) using the APDM Mobility Lab^™^ six inertial sensor system (APDM^™^; Oregon; version 1) under three conditions: (1) self-selected speed (SS), (2) fast as possible speed (FP), and (3) dual-task (DT). The DT condition involved the participant performing a verbal fluency task (Animal Naming) during the SS 2MWT. FP and DT conditions were used to create gait “stress” conditions that might amplify differences between the three movement disorders under study. Variables were selected from the five key gait domains thought to reflect independent features of neural locomotor control in older adults (30, 31), including (1) gait pace (stride length and velocity), (2) rhythm (cadence), (3) gait variability (stride length, stride velocity, and cadence variabilities), (4) gait cycle phase (percentage of gait cycle spent in double limb support and swing phases), and (5) gait asymmetry [stride length asymmetry and arm symbolic symmetry index (32)]. Stride length asymmetry was calculated as a percentage via the following formula: $\left|\frac{\left({\mathrm{stride length}}_{\mathrm{left}}-{\mathrm{stride length}}_{\mathrm{right}}\right)}{\max\;\left({\mathrm{stride length}}_{\mathrm{left}}{\mathrm{,stride length}}_{\mathrm{right}}\right)}\right|\times 100.$ Higher values of both gait asymmetry variables indicate greater asymmetry. Intra-individual gait variability was determined by the coefficient of variation ( $\frac{\mathrm{standard}\;\mathrm{deviation}}{\mathrm{mean}}\times 100)$ for each gait parameter. A movement transition domain consisting of turn duration, and number of steps to turn was also created as previously described (8) to ascertain whether these were different among the three movement disorders. The level of interference of the cognitive DT on gait performance, or the dual-task cost (DTC), was calculated as $\frac{DT-SS}{SS}\times 100$ . In addition, a validated and reliable instrumented Timed Up and Go (i-TUG) was performed six times as previously described (8) and the mean values for sit-to-stand and turn-to-sit measures were calculated.

### Cognitive assessments

Four measures of executive function were administered: the Behavioral Dyscontrol Scale II (BDS-II), the Controlled Oral Word Association Test (COWAT), the Animal Naming test, and the Symbol Digit Modalities Test (SDMT). The BDS-II is a measure of attention and inhibitory control of voluntary motor behavior (33) and the COWAT and Animal Naming tests are measures of verbal fluency (34, 35). The SDMT is a measure of attention and information processing speed (36); the oral version was used so that test results were not altered by the participants’ motor symptoms. The Wechsler Abbreviated Scale of Intelligence 3rd edition (WASI-III) was used to obtain a full intelligence quotient (Full IQ), verbal IQ (VIQ) and performance IQ (PIQ) (37). These executive function and intelligence scales were administered because there are known executive function deficits in FXTAS, PD, and ET and lower cognitive function negatively impacts gait and functional mobility in these disorders and therefore could be included as potential confounders in our statistical analysis plan. For example, lower executive function correlates with worse deficits in stride length, speed, variability and asymmetry in PD (26, 38–40), and greater impairments in velocity, cadence, stride length, and double limb support time were also associated with lower cognitive scores in ET (18). We previously found that lower information processing speed was associated with shorter stride lengths and lower response inhibition was associated with slower turn-to-sit times on the i-TUG in FXTAS (41).

### Neuropathy testing

Participants were also administered the Total Neuropathy Score (TNS), modified to exclude nerve conduction velocity testing, from a neurologist (42). Testing for neuropathy is important given that it is prevalent in FXTAS (43) and PD (44) and may negatively affect performance on spatiotemporal measures of gait (45).

### FXTAS rating scale

Participants were videotaped performing the FXTAS Rating Scale (FXTAS-RS), a 44-item scale that rates tremor, postural sway, gait, parkinsonism, coordination, dystonia, speech, and oculomotor deficits to determine the presence and severity of FXTAS symptoms (46). The scale was created using items from the Unified Parkinson’s Disease Rating Scale (UPDRS) (47), the Clinical Rating Scale for Tremor (CRST) (48), the International Cooperative Ataxia Rating Scale (ICARS) (49), and a tandem item from the Unified Huntington’s Disease Rating Scale (50). The leg agility and pouring items were not collected for all participants, therefore, only forty-two items were included in the scale. Videotapes were acquired for 16 control, 16 FXTAS, 14 PD, and 10 ET participants, which were rated by a movement disorders neurologist who was blinded to genotype.

### Molecular analysis

Blood samples or buccal swabs from all participants were sent to the Rush University Molecular Diagnostic Laboratory (Dr. Berry-Kravis lab) for *FMR1* genotype testing. QIAGEN Blood and Tissue DNA isolation kits were used to isolate DNA from buccal swabs or peripheral blood leukocytes. Allele-specific CGG repeat lengths were determined using the Asuragen Amplidex *FMR1* mPCR kit (Asuragen Inc. Austin, TX) as previously described (51).

### Statistical analysis

All measures were first compared univariately between the four participant groups with one-way ANOVA and Tukey’s *post hoc* pairwise comparisons (for normally distributed measures) or the Kruskal-Wallis test followed by pairwise comparisons with Dunn’s test for multiple comparisons (for non-normal measures). Significant gait measures from univariate comparisons were then included in a penalized multinomial logistic regression model to determine which gait measures were best able to distinguish between the groups. Sex differences were first examined within each group to determine if sex should be included as a covariate in the regression model. Age, SDMT scores, and TNS were controlled for in the final regression analysis, as some were significantly different between the groups and thought to be potentially confounding factors. There were a few sex differences in the gait and i-TUG variables within the FXTAS group, but none of these variables were significantly different between groups in the univariate comparisons and therefore sex was not included as a covariate in the final regression model. For significant logistic regression results, ROC analyses were performed and area under the curve (AUC) was computed with 95% confidence intervals for significant between group differences. Sensitivity and specificity were then calculated using the Youden index.

Spearman’s rank correlation coefficient (*rho*) was used to assess the relationship between the gait and i-TUG parameters and FXTAS-RS scores in the three movement disorder groups and between CGG repeat size in the FXTAS group. CGG repeat size did not correlate with any gait or i-TUG measures under any condition; therefore, these were not examined as potential predictors of the gait and i-TUG measures in a separate regression model in the FXTAS group. A *p* ≤ 0.05 was considered significant. Statistical analyses were performed with SAS (SAS Institute Inc., Cary NC, USA), GraphPad Prism 9 (GraphPad Software, San Diego, CA, USA), and ‘pmlr’ package in R (R Core Team 2016). For the modified FXTAS-RS, missing values were imputed using the Hot Deck technique.

## Results

### Participant characteristics

Demographic and clinical characteristics are summarized in Table 1. The study included 22 participants with FXTAS, 23 with PD, 20 with ET, and 20 controls. In the FXTAS group, six had a diagnosis of possible FXTAS, eight had probable FXTAS, and eight had definite FXTAS. The three movement disorder groups did not differ in age, although the control group was significantly younger than the PD and ET groups (*p* = 0.006 and 0.04, respectively). As expected, FXTAS participants had significantly greater CGG repeat sizes than all other groups (*p* < 0.0001) and all were in the premutation range. FXTAS and PD participants also had significantly higher TNS scores than controls (*p* = 0.0002 and 0.02, respectively) but there were no significant differences in TNS scores among the 3 movement disorders. ET participants had significantly longer disease duration compared to FXTAS and PD participants (*p* = 0.004 and 0.001, respectively). CGG repeat size did not correlate with any gait or i-TUG measures under any condition; therefore, these were not examined as predictors of gait and i-TUG measures in a separate regression model in the FXTAS group. All movement disorder groups had significantly worse FXTAS-RS scores compared to controls (*p* < 0.0001) but were not different from each other. There were no significant differences in BMI, education level, WASI Full IQ or VIQ between any of the groups. PD participants had significantly lower PIQ compared to controls (*p* = 0.04). Roughly 48, 87 and 65% of FXTAS, PD and ET participants, respectively, were on medication for motor symptoms at the time of testing (Supplementary Table S1).

**Table 1:** Participant demographic characteristics.

Variable	Controls (*n* = 20)	FXTAS (*n* = 22)	PD (*n* = 23)	ET (*n* = 20)
Age	62.65 ± 8.52 (50–83)	69.14 ± 8.12 (55–86)	71.26 ± 7.87 (56–87)a**	69.80 ± 8.85 (53–85)a*
Men, n (%)	11 (55.0)	12 (54.5)	15 (65.2)	10 (50.0)
Ethnicity, n	19 White/Non-Hispanic, 1 White/Hispanic	22 White/Non-Hispanic	20 White/Non-Hispanic, 1 White/Hispanic, 1 Asian, 1 African American	19 White/Non-Hispanic, 1 African American
BMI	27.07 ± 3.44 (20.6–35.3)	25.63 ± 4.82 (16.9–34.7)	25.94 ± 3.62 (19.5–33.8)	26.93 ± 5.39 (19.6–42.0)
Disease duration (years)	N/A	6.59 ± 4.22 (1–16)	5.74 ± 3.74 (1–15)	13.29 ± 9.97 (2–33)b**,c**
History of diabetes, n (%)	2 (10.0)	3 (13.6)	0 (0.0)	2 (10.0)
CGG repeat	31.39 ± 5.42 (23–48)	85.33 ± 12.33 (60–104)a****	29.64 ± 5.02 (20–42)b****	29.10 ± 6.17 (20–44)b****
FXTAS Dx	N/A	6 Possible, 8 Probable, 8 Definite	N/A	N/A
FXTAS-RS	13.6 ± 7.9 (3–26)	46.4 ± 17.6 (24–78)a****	41.7 ± 13.1 (21–65)a****	46.1 ± 19.6 (24–73)a****
H&Y stage	N/A	N/A	2.09 ± 0.29 (2–3)	N/A
TNS	0.59 ± 1.12 (0–4)	3.67 ± 3.02 (0–14)a***	2.95 ± 3.43 (0–13)a*	2.62 ± 2.99 (0–8)
Education	17.50 ± 2.61 (12–24)	15.95 ± 3.11 (9–20)	16.61 ± 2.69 (12–22)	15.75 ± 2.40 (12–20)
WASI full IQ	127.35 ± 9.98 (108–142)	118.13 ± 13.00 (84–135)	117.70 ± 15.24 (86–149)	116.85 ± 15.17 (84–136)
WASI VIQ	124.50 ± 8.57 (106–136)	118.07 ± 10.33 (88–129)	120.17 ± 13.45 (91–140)	117.55 ± 12.93 (86–136)
WASI PIQ	124.00 ± 11.53 (99–141)	113.53 ± 14.54 (83–141)	111.56 ± 16.69 (84–141)a*	111.65 ± 15.94 (86–134)
BDS-II	25.45 ± 1.10 (24–27)	23.33 ± 2.99 (16–27)	24.22 ± 1.93 (19–27)	24.20 ± 1.61 (21–27)
COWAT	108.20 ± 19.76 (82–142)	96.86 ± 24.98 (54–152)	101.35 ± 23.84 (74–160)	99.25 ± 23.42 (66–154)
SDMT	106.89 ± 10.05 (94–128)	89.56 ± 13.20 (67–118)a***	88.87 ± 12.84 (61–107)a****	88.95 ± 10.75 (72–103)a****
Animal naming	36.20 ± 9.56 (22–55)	28.71 ± 11.34 (11–53)	28.73 ± 8.93 (9–42)	27.40 ± 10.30 (13–48)a*

All values are mean ± SD with range in brackets unless indicated otherwise. **a, significantly different from controls; b, significantly different from FXTAS; c, significantly different from PD**; *p ≤ 0.05, **p ≤ 0.01, ***p ≤ 0.001, ****p ≤ 0.0001. Age, disease duration, CGG repeat, modified FXTAS Rating Scale score (FXTAS-RS), Hoen & Yahr (H&Y) Stage, Body Mass Index (BMI), Total Neuropathy Score (TNS), Education, Wechsler Abbreviated Scale of Intelligence (WASI) Full Intelligence Quotient (Full IQ), Verbal IQ (VIQ) and Performance IQ (PIQ), Behavioral Dyscontrol Scale II (BDS-II), Controlled Oral Word Association Test (COWAT), Symbol Digit Modalities Test (SDMT) and Animal Naming test were compared between controls, FXTAS, PD and ET. The COWAT and SDMT were scaled for age and years of education.

### Cognitive assessments

A summary of between group differences in cognitive function is also shown in Table 1. FXTAS, PD and ET participants all scored lower than controls on the SDMT (*p* = 0.0002 to <0.0001). On Animal Naming, ET subjects scored significantly lower than controls (*p* = 0.04). No significant differences were found among any of the movement disorder groups on any of the cognitive measures.

### Gait parameters

#### 2MWT

**Figure 1:**
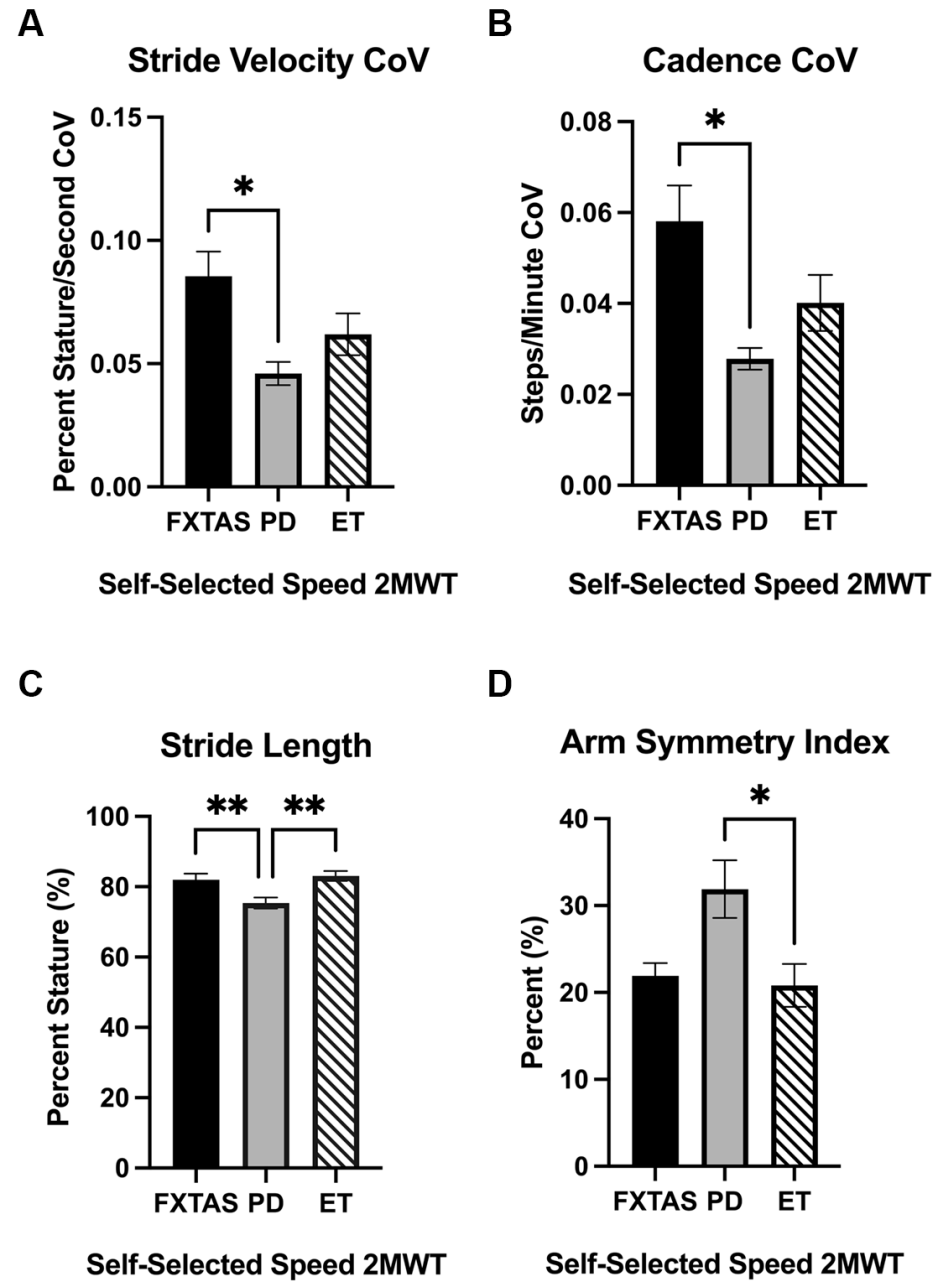
Gait parameters under self-selected (SS) speed two-minute walk test (2MWT). Significantly different gait parameters among FXTAS, PD, and ET participants: **(A)** stride velocity variability, **(B)** cadence variability, **(C)** stride length, and **(D)** arm symmetry index. $CoV\;\left(\mathrm{coefficient of variation}\right)=\frac{SD}{\mathrm{mean}}\times 100$ . All data reported as mean ± SEM. *p ≤ 0.05, **p ≤ 0.01.

**Figure 2:**
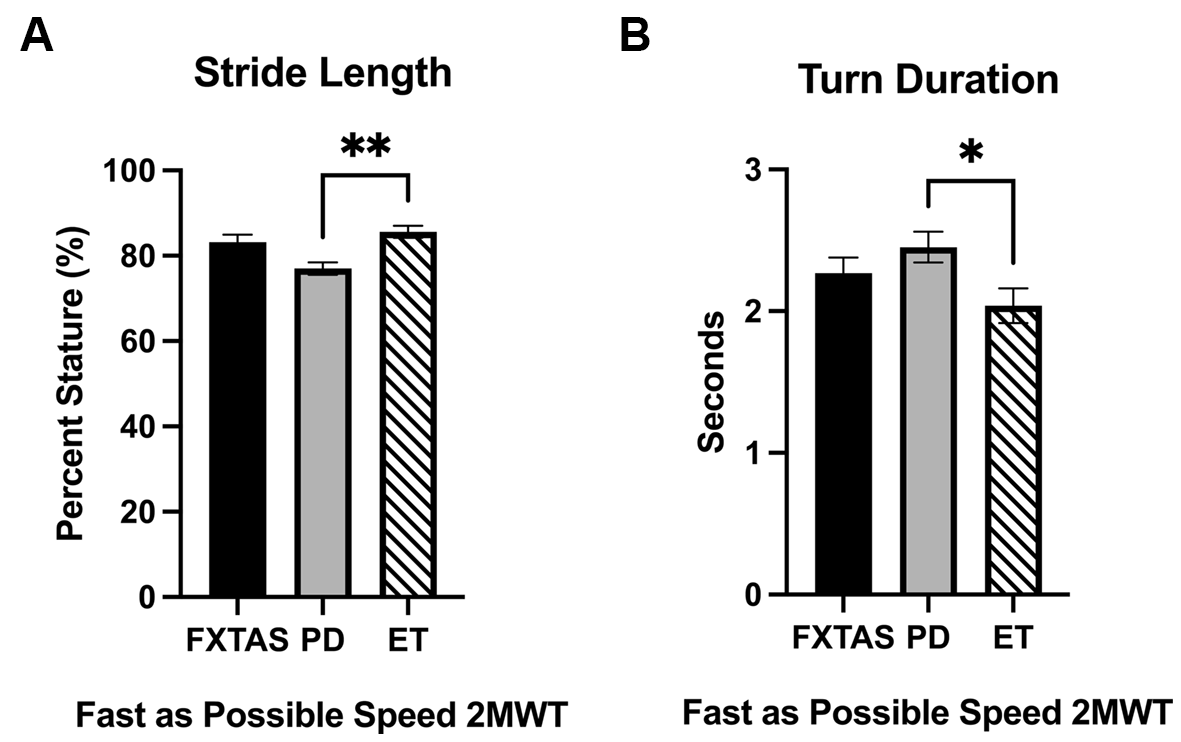
Gait parameters under fast as possible (FP) two-minute walk test (2MWT). Significantly different gait and movement transition parameters among FXTAS, PD, and ET participants: **(A)** stride length, and **(B)** turn duration. All data reported as mean ± SEM. *p ≤ 0.05, **p ≤ 0.01.

Summaries of between group comparisons of gait parameters for the three walking conditions (SS, FP, and DT) are shown in Table 2. Under the SS condition, FXTAS participants demonstrated significantly increased stride velocity variability and cadence variability compared to PD participants (*p* = 0.048 and 0.04, respectively) (Figures 1A,B). PD participants had significantly shorter stride lengths compared to FXTAS (*p* = 0.007), ET (*p* = 0.002), and control participants (*p* = <0.0001) (Figure 1C), and slower stride velocity compared to controls (*p* = 0.003). They also had significantly greater arm asymmetry than ET and control participants (*p* = 0.02 and 0.004, respectively) (Figure 1D). Lastly, PD participants took significantly longer to complete turns (*p* = 0.006), more steps to turn (*p* = 0.03), and slower peak turn velocity than control participants (*p* = 0.04). Under the FP condition, FXTAS participants had significantly slower stride velocity (*p* = 0.003), increased stride velocity variability (*p* = 0.03), increased stride length asymmetry (*p* = 0.02), and increased turn duration (*p* = 0.02) compared to controls. PD participants had significantly shorter stride lengths (*p* = 0.002 and < 0.0001, respectively) and longer turn duration (*p* = 0.03 and 0.0003, respectively) compared to ET and control participants (Figure 2). They also had slower stride velocity (*p* = 0.0002), greater arm asymmetry (*p* = 0.0005), and reduced peak turn velocity (*p* = 0.002) compared to controls. In the DT condition, FXTAS participants took significantly longer to complete turns (*p* = 0.03) and had slower peak turn velocity (*p* = 0.03) compared to controls. PD participants had significantly shorter stride lengths (*p* = 0.03, 0.02, and < 0.0001, respectively) and greater arm asymmetry (*p* = < 0.0001, 0.0002, and < 0.0001, respectively) compared to FXTAS, ET, and control participants. They also had significantly slower stride velocity (*p* < 0.0001), longer turn duration (*p* < 0.0001), slower peak turn velocity (*p* = 0.0001), and took more steps to turn (*p* = 0.009) compared to controls.

**Table 2:** Gait and turning parameters during self-selected (SS), fast as possible (FP), and dual task (DT) two-minute walk test (2MWT).

i-WALK domain parameters	Controls (*n* = 20)	FXTAS (*n* = 22)	PD (*n* = 23)	ET (*n* = 20)
Self-selected (SS)	Mean (SD)	Mean (SD)	Mean (SD)	Mean (SD)
Stride length (%stature)	86.21 (3.48)	81.93 (8.00)	75.34 (7.51)a****,b**	83.06 (6.43)c**
Stride velocity (%stature/s)	81.77 (7.09)	75.77 (8.28)	72.17 (9.27)a**	76.61 (9.84)
Cadence (steps/min)	113.62 (8.91)	110.93 (9.24)	114.97 (9.45)	110.63 (11.89)
Double limb support (%)	20.22 (3.26)	22.99 (4.90)	21.67 (4.87)	23.12 (4.96)
Trunk frontal ROM (degrees) CoV	0.35 (0.26)	0.44 (0.33)	0.24 (0.16)	0.32 (0.32)
Stride length (%stature) CoV	0.04 (0.03)	0.05 (0.02)	0.04 (0.02)	0.04 (0.02)
Stride velocity (%stature/s) CoV	0.06 (0.04)	0.08 (0.05)	0.05 (0.02)b*	0.06 (0.04)
Cadence (steps/min) CoV	0.04 (0.03)	0.06 (0.04)	0.03 (0.01)b*	0.04 (0.03)
Stride length asymmetry (%)	1.42 (0.66)	1.83 (0.71)	1.60 (0.56)	1.47 (0.60)
Arm symmetry index (%)	18.20 (5.27)	21.90 (6.78)	31.89 (15.89)a**	20.81 (11.02)c*
Turn duration (s)	2.06 (0.37)	2.43 (0.53)	2.71 (0.61)a**	2.26 (0.63)
Number of steps to turn	4.33 (0.71)	4.93 (0.97)	5.44 (1.39)a*	4.48 (0.86)
Peak turn velocity	169.70 (37.50)	150.96 (26.92)	137.54 (31.43)a*	166.97 (46.79)
Fast as possible (FP)	Mean (SD)	Mean (SD)	Mean (SD)	Mean (SD)
Stride length (%stature)	88.52 (4.22)	83.23 (7.81)	77.03 (6.99)a****	85.64 (6.39)c**
Stride velocity (%stature/s)	96.77 (9.35)	84.71 (9.90)a**	82.02 (10.54)a***	88.97 (13.27)
Cadence (steps/min)	131.12 (12.39)	122.05 (12.95)	127.85 (12.86)	124.53 (16.43)
Double limb support (%)	16.75 (3.25)	19.46 (5.49)	18.69 (4.69)	19.50 (5.15)
Trunk frontal ROM (degrees) CoV	0.36 (0.28)	0.47 (0.43)	0.27 (0.18)	0.32 (0.25)
Stride length (%stature) CoV	0.04 (0.03)	0.06 (0.03)	0.04 (0.02)	0.05 (0.03)
Stride velocity (%stature/s) CoV	0.06 (0.05)	0.09 (0.05)a*	0.05 (0.03)	0.07 (0.04)
Cadence (steps/min) CoV	0.04 (0.03)	0.06 (0.04)	0.04 (0.02)	0.05 (0.03)
Stride length asymmetry (%)	1.34 (0.41)	1.98 (0.83)a*	1.61 (0.73)	1.57 (0.67)
Arm symmetry index (%)	14.00 (6.05)	17.61 (4.42)	29.59 (17.33)a***	21.35 (11.03)
Turn duration (s)	1.81 (0.32)	2.27 (0.51)a*	2.45 (0.52)a***	2.04 (0.55)c*
Number of steps to turn	4.63 (0.67)	5.00 (0.74)	5.52 (1.26)	4.71 (0.85)
Peak turn velocity	199.34 (41.69)	167.90 (34.68)	154.06 (31.86)a**	184.33 (50.59)
Dual-task (DT)	Mean (SD)	Mean (SD)	Mean (SD)	Mean (SD)
Stride length (%stature)	86.92 (4.50)	80.88 (9.38)	73.22 (8.78)a****,b*	82.23 (6.86)c*
Stride velocity (%stature/s)	85.72 (11.99)	74.75 (11.11)	68.52 (10.25)a****	75.06 (10.91)
Cadence (steps/min)	117.95 (13.41)	110.68 (11.64)	112.52 (12.31)	109.31 (12.66)
Double limb support (%)	20.17 (3.66)	23.43 (5.91)	22.03 (4.44)	23.95 (4.73)
Trunk frontal ROM (degrees) CoV	0.38 (0.25)	0.45 (0.37)	0.27 (0.15)	0.36 (0.30)
Stride length (%stature) CoV	0.03 (0.02)	0.05 (0.03)	0.04 (0.02)	0.04 (0.03)
Stride velocity (%stature/s) CoV	0.06 (0.04)	0.08 (0.05)	0.05 (0.02)	0.07 (0.04)
Cadence (steps/min) CoV	0.04 (0.03)	0.06 (0.04)	0.03 (0.02)	0.05 (0.03)
Stride length asymmetry (%)	1.40 (0.53)	1.98 (0.86)	1.76 (0.66)	1.54 (0.55)
Arm symmetry index (%)	18.08 (5.59)	19.48 (5.63)	35.70 (19.69)a****,b****	20.03 (6.84)c***
Turn duration (s)	1.88 (0.39)	2.51 (0.86)a*	2.80 (0.69)a****	2.26 (0.66)
Number of steps to turn	4.16 (0.72)	4.98 (1.36)	5.49 (1.38)a**	4.45 (0.99)
Peak turn velocity	191.37 (42.21)	156.46 (38.28)a*	136.85 (34.44)a***	166.54 (42.53)

Gait and movement transition domain variables for between group comparisons among FXTAS, PD, ET, and controls. $CoV\;\left(\mathrm{coefficient}\;\mathrm{of}\;\mathrm{variation}\right)=\frac{SD}{\mathrm{mean}}\times 100$ . **a, significantly different from controls; b, significantly different from FXTAS; c, significantly different from PD**; *p ≤ 0.05, **p ≤ 0.01, ***p ≤ 0.001, ****p ≤ 0.0001.

#### Dual-task interference

**Figure 3:**
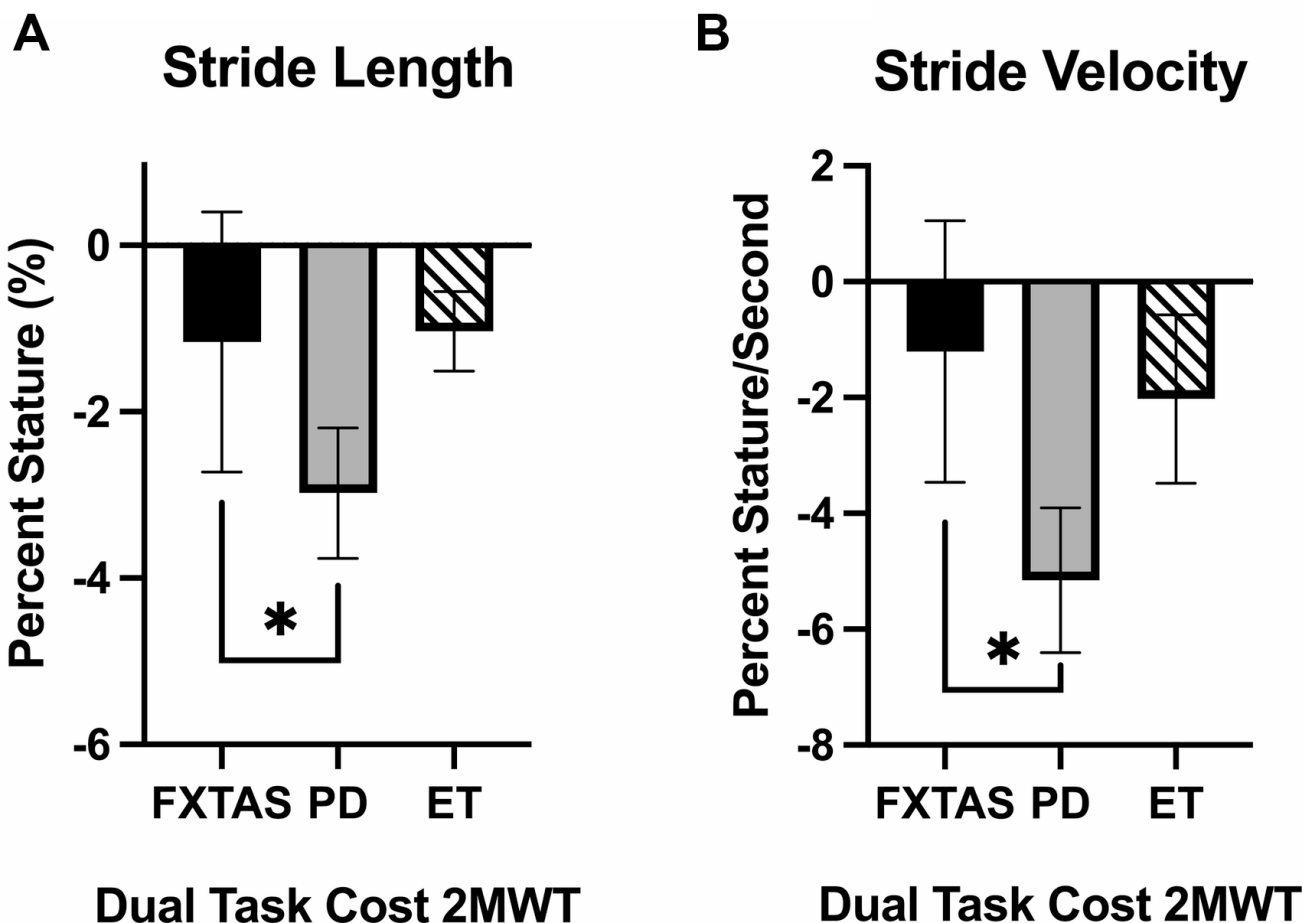
Dual task costs for gait parameters. Significantly different gait parameters among FXTAS, PD, and ET participants: **(A)** stride length, and **(B)** stride velocity. DTC was calculated using the formula $\frac{DT-SS}{SS}\times 100$ . All data reported as mean ± SEM. *p ≤ 0.05.

Dual-task costs (DTC) on 2MWT parameters are summarized in Table 3. Compared to FXTAS participants and controls, PD participants had greater DTC for stride length (*p* = 0.02 and 0.004 respectively) and stride velocity (*p* = 0.03 and 0.0006, respectively) (Figure 3). They also had greater DTC for cadence (*p* = 0.009), turn duration (*p* = 0.02), and peak turn velocity (*p* = 0.02) compared to controls. ET participants had greater DTC for peak turn velocity (*p* = 0.04) compared to controls.

**Table 3:** Dual-task costs for gait and turning parameters.

i-WALK domain parameters	Controls (*n* = 20)	FXTAS (*n* = 22)	PD (*n* = 23)	ET (*n* = 20)
Dual-task cost (DTC)	Mean (SD)	Mean (SD)	Mean (SD)	Mean (SD)
Stride length (%stature)	0.81 (2.77)	−1.16 (7.34)	−2.98 (3.76)a**,b*	−1.03 (2.14)
Stride velocity (%stature/s)	4.69 (9.68)	−1.20 (10.59)	−5.15 (5.98)a***,b*	−2.02 (6.51)
Cadence (steps/min)	3.75 (7.43)	−0.26 (5.38)	−2.23 (4.72)a**	−1.13 (5.10)
Double limb support (%)	−0.07 (10.16)	1.98 (13.63)	2.60 (8.96)	4.29 (9.85)
Trunk frontal ROM (degrees) CoV	24.66 (47.14)	4.07 (31.88)	18.10 (34.02)	22.04 (32.84)
Stride length (%stature) CoV	3.28 (50.80)	3.58 (38.33)	8.15 (33.95)	3.62 (28.48)
Stride velocity (%stature/s) CoV	11.68 (55.87)	7.97 (37.26)	15.20 (36.37)	19.94 (44.09)
Cadence (steps/min) CoV	31.75 (70.57)	16.33 (51.26)	25.47 (35.78)	30.96 (56.67)
Stride length asymmetry (%)	5.84 (37.59)	15.58 (58.73)	11.49 (24.48)	7.56 (22.96)
Arm symmetry index (%)	2.40 (34.04)	−7.26 (25.27)	17.06 (59.66)	7.28 (37.98)
Turn duration (s)	−7.84 (13.04)	3.99 (32.21)	3.74 (12.17)a*	0.64 (12.43)
Number of steps to turn	−2.73 (14.55)	1.94 (23.55)	1.52 (10.83)	−0.49 (11.34)
Peak turn velocity	13.19 (13.19)	3.77 (17.93)	0.04 (14.79)a*	0.88 (10.64)a*

Dual-task costs (DTC) for gait variables for between group comparisons among FXTAS, PD, ET, and controls. DTC was calculated using the formula $\frac{DT-SS}{SS}\times 100$ . $CoV\;\left(\mathrm{coefficient}\;\mathrm{of}\;\mathrm{variation}\right)=\frac{SD}{\mathrm{mean}}\times 100$ . **a, significantly different from controls; b, significantly different from FXTAS**; *p ≤ 0.05, **p ≤ 0.01, ***p ≤ 0.001, ****p ≤ 0.0001.

#### i-TUG

**Figure 4:**
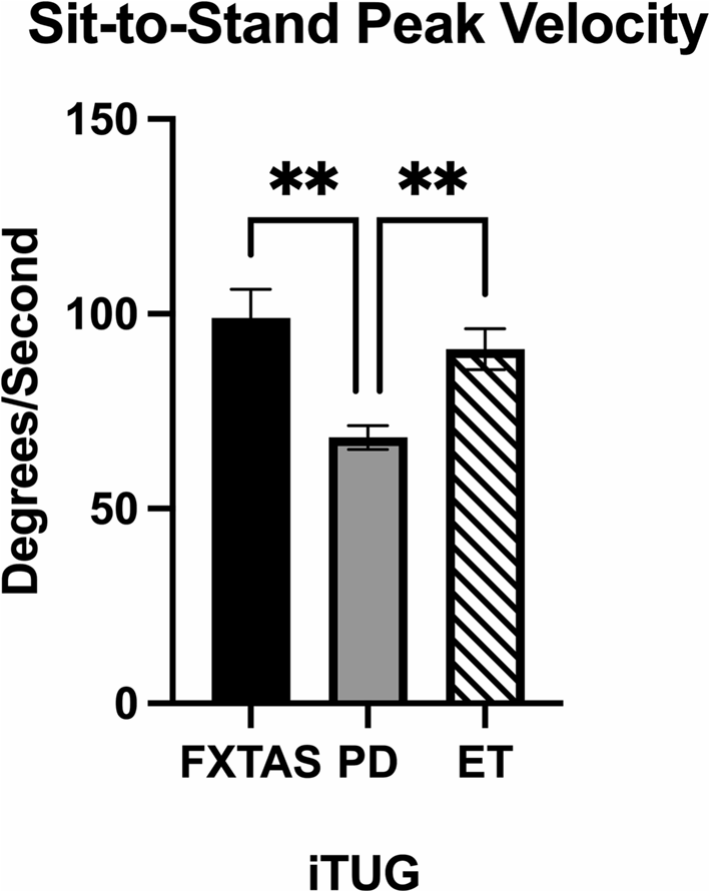
Movement transition parameters in the Instrumented Timed Up and Go test (i-TUG). Significantly different parameter among FXTAS, PD, and ET participants: sit-to-stand peak velocity. All data reported as mean ± SEM. *p ≤ 0.05, **p ≤ 0.01.

Summaries of between group comparisons of i-TUG parameters are summarized in Table 4. PD participants had significantly reduced sit-to-stand peak velocity compared to FXTAS (*p* = 0.002), ET (*p* = 0.009) and control participants (*p* = 0.007) (Figure 4), and reduced turn-to-sit peak turn velocity compared to controls (*p* = 0.006).

**Table 4:** Movement transition parameters during the Instrumented Timed Up and Go test (i-TUG).

i-TUG parameters	Controls (*n* = 20)	FXTAS (*n* = 22)	PD (*n* = 23)	ET (*n* = 20)
	Mean (SD)	Mean (SD)	Mean (SD)	Mean (SD)
Total duration (s)	17.71 (2.28)	20.54 (5.47)	20.02 (2.64)	18.95 (3.69)
Sit-to-stand duration (s)	2.28 (0.30)	2.34 (0.26)	2.49 (0.23)	2.35 (0.32)
Sit-to-stand peak velocity (deg/s)	99.29 (47.45)	98.91 (33.63)	68.26 (14.90)a**,b**	90.95 (23.46)c**
Turn-to-sit peak turn velocity (deg/s)	173.98 (39.34)	151.89 (36.32)	135.15 (36.34)a**	160.50 (38.74)
Turn-to-sit duration (s)	4.21 (0.65)	4.57 (1.03)	4.56 (0.98)	4.26 (0.82)

i-TUG variables for between group comparisons among FXTAS, PD, ET, and controls. **a, significantly different from controls; b, significantly different from FXTAS; c, significantly different from PD**; *p ≤ 0.05, **p ≤ 0.01, ***p ≤ 0.001, ****p ≤ 0.0001.

#### Regression analysis

**Figure 5:**
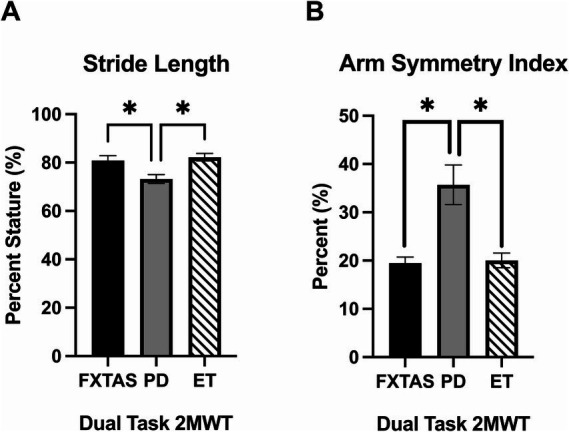
Significant multinomial regression results. Significantly different gait parameters among FXTAS, PD, and ET participants during the dual task (DT) condition: **(A)** stride length, and **(B)** arm symmetry index. All data reported as mean ± SEM. *p ≤ 0.05.

In the multinomial logistic regressions controlling for age, SDMT, and TNS, stride length on the DT 2MWT was able to distinguish PD from FXTAS (OR = 0.88, 95% CI = 0.77–0.996, *p* = 0.04) and ET (OR = 1.16, 95% CI = 1.02–1.33, *p* = 0.02), such that participants with shorter stride length were more likely to have a diagnosis of PD (Figure 5A). Arm symmetry index during the DT 2MWT was also able to distinguish between PD from FXTAS (OR = 1.1, 95% CI = 1.01–1.19, *p* = 0.03) and ET (OR = 0.92, 95% CI = 0.85–1.00, *p* = 0.05), such that participants with greater arm asymmetry were more likely to be diagnosed with PD (Figure 5B). No gait or i-TUG variables were found to distinguish FXTAS from ET. Given the relatively low group sample sizes in this study, a multivariable ROC analysis was performed accounting for age, TNS, SDMT scores, stride length during DT gait, and arm symmetry index during DT gait. The ROC analysis comparing FXTAS and PD groups had an AUC of 0.85 (95% CI: 0.73–0.97) with a sensitivity of 0.83 and specificity of 0.71 based on the Youden index. The comparison between PD and ET resulted in an AUC of 0.87 (95% CI: 0.76–0.98) with a sensitivity of 0.81 and specificity of 0.81.

#### Correlations

Spearman’s correlations between FXTAS-RS scores and gait parameters are summarized in Supplementary Tables S2–S5. Under the SS condition, worse (higher) FXTAS-RS scores were associated with reduced stride length (*p* = 0.03), stride velocity (*p* = 0.003), and peak turn velocity (*p* = 0.006), as well as increased arm asymmetry (*p* = 0.03), turn duration (*p* = 0.003), and number of steps to turn (*p* = 0.03) in FXTAS. On the FP 2MWT, higher FXTAS-RS scores were associated with reduced stride velocity (*p* = 0.005), cadence (*p* = 0.048) and peak turn velocity (*p* = 0.02), as well as increased turn duration (*p* = 0.005) in FXTAS, and lower steps to turn in controls (*p* = 0.03). During the DT 2MWT, worse FXTAS-RS scores were associated with greater stride length asymmetry (*p* = 0.04) and turn duration (*p* = 0.047), as well as reduced stride length (*p* = 0.04), stride velocity (*p* = 0.002), and peak turn velocity (*p* = 0.02) in FXTAS. No significant correlations were found between FXTAS-RS and spatiotemporal variables of gait and turning in PD or ET during SS, FP, or DT walking. On the i-TUG, higher FXTAS-RS scores were correlated with increased total duration in PD (*p* = 0.01) and increased turn-to-sit duration in both PD (*p* = 0.02) and ET (*p* = 0.02). In FXTAS participants, no significant correlations were found between CGG repeat size and gait or i-TUG variables.

## Discussion

This is the first study to directly compare gait characteristics in FXTAS, PD, and ET using quantitative gait analysis and gait stress tests including FP and DT paradigms. These results show that gait analysis was able to distinguish between-group differences in gait parameters during SS, FP, and DT conditions. We also identified differences in DTC in the domains of gait pace (stride length and velocity) where PD had significant DTC compared to FXTAS participants. FXTAS participants had significantly slower stride velocity and stride velocity variability, increased stride length asymmetry, and increased turn duration compared to controls during FP walking. Importantly, this condition revealed a greater number of impairments in FXTAS than in ET compared to controls, suggesting that this particular test may be helpful for distinguishing FXTAS from ET in the clinic. It is known that at fast speeds of locomotion, it is more difficult to maintain stability due to signaling delays between the musculoskeletal system and higher-level neural control centers (52). It is possible that this coordination of neural signaling and muscular responses was more stressed by fast walking in the FXTAS participants, requiring them to slow down their strides and turns in order to maintain stability more so than the ET participants. In a previous study, our group characterized the gait deficits using a 7 m i-TUG in a smaller cohort of FXTAS participants and found abnormalities similar to those found in this study, including reduced stride velocity and longer turn duration (8); however, these deficits were seen with SS walking speeds, whereas the current study did not find any gait deficits in FXTAS compared to controls at these speeds. Our present inclusion criteria required that participants had to be able to walk unassisted for 2 min; therefore, the group had milder gait symptoms that might not be detectable at SS speeds. In addition, our prior study only included those with definite cerebellar gait ataxia on neurological exam, whereas the current study included a more heterogeneous group of FXTAS participants with both tremor and ataxia dominant forms of the disease.

Other groups have investigated gait under fast walking speeds in other cerebellar ataxias and reported increased stride length and speed variability in Friedreich ataxia, spinocerebellar ataxia, and idiopathic cerebellar patients using the GAITRite^®^ walkway (53–55). Increased stride velocity variability was seen in FXTAS during FP walking in the current study, which we reported in our prior FP gait study in FXTAS to be significantly associated with increased falls (20). Furthermore, Schniepp et al. found FP walking to be the most strongly correlated to clinical severity of ataxia compared to other walking speeds and concluded that it may be a useful measure in the clinical evaluation of patients with cerebellar ataxia (55). Given that we found the most gait deficits in FXTAS under FP walking in the present study, this test may be useful for evaluating FXTAS patients in the clinic.

PD participants had significantly reduced stride length compared to FXTAS and ET participants on the SS and DT gait conditions, as well as slower stride velocity and reduced stride length compared to controls on all three conditions. They also took significantly longer to turn with lower peak turn velocity and increased turn duration on all three gait conditions, and more steps to turn under SS and DT walking compared to controls. In the FP condition, PD participants were slower to turn than the ET group. Typical PD patients display a slow, shuffling gait pattern, as well as bradykinesia, which is consistent with our findings of slower, shorter strides and slower turns. This contrast with the wide-based, ataxic gait pattern typically seen in FXTAS patients, and the mild ataxia seen in roughly half of ET patients (56–60). PD participants also had significantly greater arm asymmetry than ET participants and controls at SS speeds, and than FXTAS, ET, and control participants under DT gait. DT during gait apparently stressed the neuromotor system in PD exacerbating arm asymmetry. These results are consistent with the common asymmetric PD gait pattern. PD symptoms typically present asymmetrically and many patients exhibit a reduced or absent reciprocal arm swing (61).

ET participants were not abnormal on any gait parameters for any of the test conditions, likely because regular bipedal gait in ET patients tends to be normal (58). However, mild gait and postural stability deficits have been found in ET, including difficulties with tandem gait (56–60). It may be that the present study conditions were not challenging enough to extract gait deficits in the ET group, or that the pool of selected ET participants did not have cerebellar gait ataxia.

Compared to FXTAS participants and controls, PD participants had significantly greater DTC for the gait pace domain including stride velocity and stride length parameters. Previous DT studies in PD have shown similar findings. Plotnik et al. found that gait speed and stride length were both impaired by DT using a serial subtraction cognitive interference task (26). Yogev-Seligmann et al. and Fuller et al. also found reduced gait speed under DT in PD using a verbal fluency interference task similar to the current study (27, 62). However, we did not find differences for DTC between movement disorder groups in any of the other gait domains. None of the groups performed worse on the cognitive task during the DT condition, indicating that they were not prioritizing the gait task over the cognitive task. It is possible that the DT verbal fluency test did not provide a sufficient cognitive load to reveal other impairments. Therefore, future studies could utilize a more difficult task that might cause greater cognitive interference. Our results do suggest that PD patients may be more sensitive to cognitive interference, potentially having lower cognitive reserve than those with FXTAS.

The i-TUG was used to evaluate functional movement transitions important in daily living. PD participants had significantly slower speed when transitioning from sit-to-stand compared to FXTAS, ET, and control participants, as well as slower speed when turning to sit compared to controls. Given that bradykinesia is a cardinal symptom of PD, it was expected that the PD group would be slower at completing these movement transitions. These results suggest that the sit-to-stand measure may be helpful for assisting with diagnosis, such that patients with reduced velocities on this parameter may be more likely to have PD. Furthermore, Herman et al. found that i-TUG parameters were able to distinguish between the postural instability and gait disorder and tremor dominant subtypes of PD (10). It has been proposed that there may be two subtypes of FXTAS as well, including tremor and ataxia predominant phenotypes (63). As a follow-up study, it would be interesting to compare these subtypes of FXTAS to see if i-TUG, FP, and DT gait testing are able to distinguish them; treatment plans could then be tailored based on individual phenotypes.

Multinomial logistic regression analysis showed that on the DT condition, stride lengths was able to distinguish PD from FXTAS and ET, such that participants with shorter stride length were more likely to have PD. Shortening of steps when walking is a common feature in PD, particularly under a stressful condition such as walking while performing a cognitive task where PD patients may be triggered to festinate or take involuntarily short steps (64, 65). Furthermore, in the current study we found that PD participants had a significant DTC for stride length whereas FXTAS participants did not. Thus, it is logical that the measure of stride length would be able to make this distinction. Arm symmetry index during the DT condition was also able to distinguish between FXTAS and PD, and ET and PD, such that participants with greater arm asymmetry were more likely to have PD. This appears logical given that reduction in reciprocal arm swing range of motion and its asymmetry is a hallmark feature in PD (61), while arm asymmetry in FXTAS or ET has not been reported. Our findings of greater arm asymmetry in PD compared to ET are similar to those in a recent report using inertial sensors during performance of the i-TUG and a machine learning approach to distinguish early-stage PD from ET (66). We also have unpublished data in larger cohorts indicating that arm asymmetry and arm range of motion are not different from controls in FXTAS.

As expected, the FXTAS, PD, and ET groups all had significantly worse FXTAS-RS scores compared to healthy controls. However, no differences in rating scale scores were found among the disorders, suggesting that the gait and functional movement transition measures were more sensitive for distinguishing between them than the scale. FXTAS-RS scores were associated with multiple gait measures in FXTAS under all three gait conditions, and number of steps to turn in controls during FP walking. FXTAS-RS scores were also associated with total duration in PD and turn-to-sit duration in PD and ET during the i-TUG. This finding was not unexpected given that FXTAS and PD patients tend to have greater gait impairments than ET patients (67).

Strengths of this study include objective gait measurement using highly sensitive quantitative analysis that has been validated in PD in previous studies, and the use of DT cognitive-motor interference paradigms similar to those used in previous studies of PD, FXTAS, and ET. SS, FP, and DT gait testing was able to distinguish differences between FXTAS and PD and ET and PD. It may be cost effective to add these tests to a clinical evaluation to aid in accurate diagnosis given that each walking condition takes only 2 min to complete. These quantitative measures may improve characterization of these disorders and serve as outcome measures to evaluate treatment responses in future studies.

Limitations of this pilot study include a relatively small sample size; increasing the sample size in future studies will help to strengthen and corroborate these findings. Another limitation is that there were no significant differences between controls and FXTAS participants on any gait or turn variables in the self-selected (SS) speed condition, suggesting that our FXTAS group was minimally impaired in gait and only showed impairments at fast speeds (FP) and while dual tasking (DT). Future studies could only include those with probable and definite FXTAS with definite cerebellar gait ataxia on clinical exam. The control group was significantly younger than the PD and ET groups, but there were no differences in age between the three movement disorder groups. Additionally, we controlled for age in the regression model, which only compared FXTAS, PD and ET groups, so we do not believe age is a relevant problem with the study. Another potential limitation was that, due to logistical and feasibility issues, all medicated study participants were on their medications at time of testing, which did not allow their gait to be measured in its most natural and debilitating state. In future studies, it would be ideal if participants could be tested both on and off their medication to obtain a more accurate measurement of gait in these disorders.

These findings demonstrate that patients with FXTAS and ET exhibit distinct gait profiles from those with PD. The DT condition was sensitive for distinguishing FXTAS and ET from PD in arm asymmetry and stride length. Significant DT cognitive interference (i.e., DTC) for gait and turn variables were only seen in the PD group. On the i-TUG, FXTAS and ET participants were significantly faster at transitioning from sitting to standing than PD participants. These results suggest that DT walking paradigms and assessment of movement transitions may be useful for diagnosing FXTAS patients in the clinic.

## Data availability statement

The raw data supporting the conclusions of this article will be made available by the authors, without undue reservation.

## Ethics statement

The studies involving humans were approved by the Rush University Institutional Review Board. The studies were conducted in accordance with the local legislation and institutional requirements. The participants provided their written informed consent to participate in this study.

## Author contributions

ER-D: Writing – original draft, Conceptualization, Data curation, Formal analysis, Funding acquisition, Investigation, Methodology, Project administration, Writing – review & editing. ET: Writing – original draft, Writing – review & editing, Data curation, Formal analysis, Visualization. GP: Writing – review & editing, Data curation, Methodology. BO: Writing – review & editing, Formal analysis. YL: Writing – review & editing, Formal analysis. EB-K: Writing – review & editing, Conceptualization. DH: Writing – review & editing, Conceptualization, Investigation, Methodology, Supervision. JO’K: Writing – original draft, Writing – review & editing, Conceptualization, Formal analysis, Funding acquisition, Investigation, Methodology, Project administration, Supervision.

## References

[1] Hagerman RJ Leehey MA Heinrichs WL Tassone F Wilson RL Hills J. Intention tremor, parkinsonism, and generalized brain atrophy in male carriers of fragile X. Neurology. (2001) 57:127–30. doi: 10.1212/wnl.57.1.127, PMID: 11445641

[2] Grigsby J Cornish K Hocking DR Kraan C Olichney J Rivera SM. The cognitive neuropsychological phenotype of carriers of the FMR1 premutation. J Neurodev Disord. (2014) 6:28. doi: 10.1186/1866-1955-6-28, PMID: 25136377 PMC4135346

[3] Seritan AL Nguyen DV Farias SET Hinton WL Grigsby J Bourgeois JA. Dementia in fragile X-associated tremor/ataxia syndrome (FXTAS): comparison with Alzheimer’s disease. Am J Med Genet. (2008) 147B:1138–44. doi: 10.1002/ajmg.b.30732, PMID: 18384046 PMC2898561

[4] Berry-Kravis E Abrams L Coffey S Hall DA Greco C Gane LW. Fragile X-associated tremor/ataxia syndrome: clinical features, genetics, and testing guidelines. Mov Disord. (2007) 22:2018–30. doi: 10.1002/mds.21493, PMID: 17618523

[5] Leehey MA. Fragile X-associated tremor/ataxia syndrome: clinical phenotype, diagnosis, and treatment. J Investig Med. (2009) 57:830–6. doi: 10.2310/jim.0b013e3181af59c4, PMID: 19574929 PMC2787702

[6] Hall DA Berry-Kravis E Jacquemont S Rice C Cogswell JB Zhang L. Initial diagnoses given to persons with the fragile X associated tremor/ataxia syndrome (FXTAS). Neurology. (2005) 65:299–301. doi: 10.1212/01.wnl.0000168900.86323.9c, PMID: 16043804

[7] Robertson E Hall DA Pal G Ouyang B Liu Y Joyce J. Tremorography in fragile X-associated tremor/ataxia syndrome, Parkinson’s disease and essential tremor. Clin Parkinsonism Relat Disord. (2020) 3:100040. doi: 10.1016/j.prdoa.2020.100040, PMID: 34316626 PMC8298795

[8] O’Keefe JA Robertson-Dick E Hall DA Berry-Kravis E. GAIT and functional mobility deficits in fragile X-associated tremor/ataxia syndrome. Cerebellum. (2015) 15:475–82. doi: 10.1007/s12311-015-0714-4, PMID: 26298472 PMC8797146

[9] Dewey DC Miocinovic S Bernstein IH Khemani P Dewey RB Querry RG. Automated gait and balance parameters diagnose and correlate with severity in Parkinson disease. J Neurol Sci. (2014) 345:131–8. doi: 10.1016/j.jns.2014.07.026, PMID: 25082782 PMC4177980

[10] Herman T Rosenberg-Katz K Jacob Y Giladi N Hausdorff JM. Gray matter atrophy and freezing of gait in Parkinson’s disease: is the evidence black-on-white? Mov Disord. (2013) 29:134–9. doi: 10.1002/mds.25697, PMID: 24151091

[11] Van Uem JMT Walgaard S Ainsworth E Hasmann SE Heger T Nussbaum S. Quantitative timed-up-and-go parameters in relation to cognitive parameters and health-related quality of life in mild-to-moderate Parkinson’s disease. PLoS One. (2016) 11:e0151997. doi: 10.1371/journal.pone.0151997, PMID: 27055262 PMC4824446

[12] King LA Mancini M Priest KC Salarian A Rodrigues-De-Paula F Horak FB. Do clinical scales of balance reflect turning abnormalities in people with Parkinson’s disease? J Neurol Phys Ther. (2012) 36:25–31. doi: 10.1097/npt.0b013e31824620d1, PMID: 22333919 PMC3290336

[13] Zampieri C Salarian A Carlson-Kuhta P Aminian K Nutt JG Horak FB. The instrumented timed up and go test: potential outcome measure for disease modifying therapies in Parkinson’s disease. J Neurol Neurosurg Psychiatry. (2009) 81:171–6. doi: 10.1136/jnnp.2009.173740, PMID: 19726406 PMC3065923

[14] Salarian A Horak FB Zampieri C Carlson-Kuhta P Nutt JG Aminian K. ITUG, a sensitive and reliable measure of mobility. IEEE Trans Neural Syst Rehabil Eng. (2010) 18:303–10. doi: 10.1109/tnsre.2010.2047606, PMID: 20388604 PMC2922011

[15] Zampieri C Salarian A Carlson-Kuhta P Nutt JG Horak FB. Assessing mobility at home in people with early Parkinson’s disease using an instrumented timed up and go test. Parkinsonism Relat Disord. (2011) 17:277–80. doi: 10.1016/j.parkreldis.2010.08.001, PMID: 20801706 PMC2995832

[16] Nelson AJ Zwick D Brody S Doran C Pulver L Rooz G. The validity of the GaitRite and the functional ambulation performance scoring system in the analysis of Parkinson gait1. NeuroRehabilitation. (2002) 17:255–62. doi: 10.3233/nre-2002-17312, PMID: 12237507

[17] Rao AK Gillman AE Louis ED. Quantitative gait analysis in essential tremor reveals impairments that are maintained into advanced age. Gait Posture. (2011) 34:65–70. doi: 10.1016/j.gaitpost.2011.03.013, PMID: 21478017 PMC3575132

[18] Rao AK Uddin J Gillman AE Louis ED. Cognitive motor interference during dual-task gait in essential tremor. Gait Posture. (2013) 38:403–9. doi: 10.1016/j.gaitpost.2013.01.006, PMID: 23369662 PMC3679258

[19] Louis ED Galecki M Rao AK. Four essential tremor cases with moderately impaired gait: how impaired can gait be in this disease? Tremor Other Hyperkinet Mov. (2013) 3:tre-03-200-4597-1. doi: 10.7916/D8QV3K7G PMC3822047 24255798

[20] O’Keefe JA Guan J Robertson E Biskis A Joyce J Ouyang B. The effects of dual task cognitive interference and fast-paced walking on gait, turns, and falls in men and women with FXTAS. Cerebellum. (2020) 20:212–21. doi: 10.1007/s12311-020-01199-3, PMID: 33118140 PMC8005408

[21] Morris ME Iansek R Matyas TA Summers JJ. Ability to modulate walking cadence remains intact in Parkinson’s disease. J Neurol Neurosurg Psychiatry. (1994) 57:1532–4. doi: 10.1136/jnnp.57.12.1532, PMID: 7798986 PMC1073238

[22] Bayle N Patel A Crisan D Guo LJ Hutin E Weisz DJ. Contribution of step length to increase walking and turning speed as a marker of Parkinson’s disease progression. PLoS One. (2016) 11:e0152469. doi: 10.1371/journal.pone.0152469, PMID: 27111531 PMC4844147

[23] Peterson DS Mancini M Fino PC Horak FB Smulders K. Speeding up gait in Parkinson’s disease. J Parkinsons Dis. (2020) 10:245–53. doi: 10.3233/jpd-191682, PMID: 31561384 PMC7304052

[24] Plotnik M Giladi N Hausdorff JM. Bilateral coordination of gait and Parkinson’s disease: the effects of dual tasking. J Neurol Neurosurg Psychiatry. (2009) 80:347–50. doi: 10.1136/jnnp.2008.157362, PMID: 19228674

[25] Vervoort G Heremans E Bengevoord A Strouwen C Nackaerts E Vandenberghe W. Dual-task-related neural connectivity changes in patients with Parkinson’ disease. Neuroscience. (2016) 317:36–46. doi: 10.1016/j.neuroscience.2015.12.056, PMID: 26762801

[26] Plotnik M Dagan Y Gurevich T Giladi N Hausdorff JM. Effects of cognitive function on gait and dual tasking abilities in patients with Parkinson’s disease suffering from motor response fluctuations. Exp Brain Res. (2010) 208:169–79. doi: 10.1007/s00221-010-2469-y, PMID: 21063692

[27] Yogev-Seligmann G Hausdorff JM Giladi N. Do we always prioritize balance when walking? Towards an integrated model of task prioritization. Mov Disord. (2012) 27:765–70. doi: 10.1002/mds.24963, PMID: 22419512

[28] Bove M Marinelli L Avanzino L Marchese R Abbruzzese G. Posturographic analysis of balance control in patients with essential tremor. Mov Disord. (2006) 21:192–8. doi: 10.1002/mds.20696, PMID: 16161140

[29] Hoehn MM Yahr MD. Parkinsonism: onset, progression, and mortality. Neurology. (1967) 17:427. doi: 10.1212/wnl.17.5.427 6067254

[30] Hollman JH McDade E Petersen RC. Normative spatiotemporal gait parameters in older adults. Gait Posture. (2011) 34:111–8. doi: 10.1016/j.gaitpost.2011.03.024, PMID: 21531139 PMC3104090

[31] Lord S Galna B Rochester L. Moving forward on gait measurement: toward a more refined approach. Mov Disord. (2013) 28:1534–43. doi: 10.1002/mds.2554, PMID: 24132841

[32] Sant’anna AP Salarian A Wickström N. A new measure of movement symmetry in early Parkinson’s disease patients using symbolic processing of inertial sensor data. IEEE Trans Biomed Eng. (2011) 58:2127–35. doi: 10.1109/tbme.2011.2149521, PMID: 21536527

[33] Luria AR. The problem of localization of functions in the cerebral cortex. Boston, MA: Springer. (1966). p. 5–38.

[34] Borkowski JG Benton AL Spreen O. Word fluency and brain damage. Neuropsychologia. (1967) 5:135–40. doi: 10.1016/0028-3932(67)90015-2

[35] Goodglass H Kaplan E. The assessment of aphasia and related disorders. (1972). Available at: https://ci.nii.ac.jp/ncid/BA24232886

[36] Smith A. Symbol Digit Modalities test. PsycTESTS Dataset. (1973). doi: 10.1037/t27513-000

[37] Wechsler D. Wechsler abbreviated scale of intelligence. PsycTESTS Dataset. (1999). doi: 10.1037/t15170-000

[38] Stegemöller EL Nocera J Malaty IA Shelley M Okun MS Hass CJ. Timed up and go, cognitive, and quality-of-life correlates in Parkinson’s disease. Arch Phys Med Rehabil. (2014) 95:649–55. doi: 10.1016/j.apmr.2013.10.031, PMID: 24291596

[39] Yogev G Giladi N Peretz C Springer S Simon ES Hausdorff JM. Dual tasking, gait rhythmicity, and Parkinson’s disease: which aspects of gait are attention demanding? Eur J Neurosci. (2005) 22:1248–56. doi: 10.1111/j.1460-9568.2005.04298.x, PMID: 16176368

[40] Rochester L Nieuwboer A Baker K Hetherington V Willems AM Kwakkel G. Walking speed during single and dual tasks in Parkinson’s disease: which characteristics are important? Mov Disord. (2008) 23:2312–8. doi: 10.1002/mds.22219, PMID: 18816800

[41] O’Keefe JA Robertson E Ouyang B Carns D McAsey A Liu Y. Cognitive function impacts gait, functional mobility and falls in fragile X-associated tremor/ataxia syndrome. Gait Posture. (2018) 66:288–93. doi: 10.1016/j.gaitpost.2018.09.005, PMID: 30243213 PMC6342509

[42] Cornblath DR Chaudhry V Carter K Lee D Seysedadr M Miernicki M. Total neuropathy score: validation and reliability study. Neurology. (1999) 53:1660. doi: 10.1212/wnl.53.8.1660 10563609

[43] Soontarapornchai K Maselli RA Fenton-Farrell G Tassone F Hagerman PJ Hessl D. Abnormal nerve conduction features in fragile X premutation carriers. Arch Neurol. (2008) 65:495–8. doi: 10.1001/archneur.65.4.495, PMID: 18413472 PMC2888466

[44] Zis P Grünewald RA Chaudhuri R Hadjivassiliou M. Peripheral neuropathy in idiopathic Parkinson’s disease: a systematic review. J Neurol Sci. (2017) 378:204–9. doi: 10.1016/j.jns.2017.05.023, PMID: 28566165

[45] De Camargo MR Barela JA Nozabieli AJL Mantovani AM Martinelli A Fregonesi CEPT. Balance and ankle muscle strength predict spatiotemporal gait parameters in individuals with diabetic peripheral neuropathy. Diabetes Metab Syndr. (2015) 9:79–84. doi: 10.1016/j.dsx.2015.02.004, PMID: 25813140

[46] Leehey MA Berry-Kravis E Goetz CG Zhang L Hall DA Li L. FMR1 CGG repeat length predicts motor dysfunction in premutation carriers. Neurology. (2007) 70:1397–402. doi: 10.1212/01.wnl.0000281692.98200.f5, PMID: 18057320 PMC2685188

[47] Fahn S. Members of the UPDRS development committee. Unified Parkinson’s disease rating scale. Recent Dev Parkinson’s Dis. (1987) 2:293–304.

[48] Fahn S Tolosa E Marin C Jankovic J. Clinical rating scale for tremor In: Jankovic J Tolosa E, editors. Parkinson’s disease and movement disorders. Baltimore, MD: Williams & Wilkins (1993). 271–80.

[49] Trouillas P Takayanagi T Hallett M Currier RD Subramony SH Wessel K. International cooperative ataxia rating scale for pharmacological assessment of cerebellar syndrome. J Neurol Sci. (1997) 145:205–11. doi: 10.1016/s0022-510x(96)00231-6, PMID: 9094050

[50] Kieburtz K Penney JB Corno P Ranen NG Shoulson I Feigin A. Unified Huntington’s disease rating scale: reliability and consistency. Mov Disord. (1996) 11:136–42. doi: 10.1002/mds.870110204, PMID: 8684382

[51] Chen L Hadd AG Sah S Houghton JD Filipovic-Sadic S Zhang W. High-resolution methylation polymerase chain reaction for fragile X analysis: evidence for novel FMR1 methylation patterns undetected in southern blot analyses. Genet Med. (2011) 13:528–38. doi: 10.1097/gim.0b013e31820a780f, PMID: 21430544 PMC4043840

[52] Nordin AD Rymer WZ Biewener AA Schwartz AB Chen D Horak FB. Biomechanics and neural control of movement, 20 years later: what have we learned and what has changed? J Neuroeng Rehabil. (2017) 14:91. doi: 10.1186/s12984-017-0298-y, PMID: 28893279 PMC5594571

[53] Stephenson JL Zesiewicz TA Gooch CL Wecker L Sullivan KL Jahan I. Gait and balance in adults with Friedreich’s ataxia. Gait Posture. (2015) 41:603–7. doi: 10.1016/j.gaitpost.2015.01.002, PMID: 25662043

[54] Schniepp R Wuehr M Neuhaeusser M Kamenova M Dimitriadis K Klopstock T. Locomotion speed determines gait variability in cerebellar ataxia and vestibular failure. Mov Disord. (2011) 27:125–31. doi: 10.1002/mds.23978, PMID: 21997342

[55] Schniepp R Strupp M Wuehr M Jahn K Dieterich M Brandt T. Acetyl-DL-leucine improves gait variability in patients with cerebellar ataxia—a case series. Cerebellum Ataxias. (2016) 3:8. doi: 10.1186/s40673-016-0046-2, PMID: 27073690 PMC4828858

[56] Singer C Sanchez-Ramos J Weiner WJ. Gait abnormality in essential tremor. Mov Disord. (2004) 9:193–6. doi: 10.1002/mds.870090212 8196682

[57] Hubble J Busenbark K Pahwa R Lyons KE Koller WC. Clinical expression of essential tremor: effects of gender and age. Mov Disord. (1997) 12:969–72. doi: 10.1002/mds.870120620, PMID: 9399222

[58] Stolze H Petersen G Raethjen J Wenzelburger R Deuschl G. The gait disorder of advanced essential tremor. Brain. (2001) 124:2278–86. doi: 10.1093/brain/124.11.2278 11673328

[59] Kronenbuerger M Konczak J Ziegler W Buderath P Frank B Coenen VA. Balance and motor speech impairment in essential tremor. Cerebellum. (2009) 8:389–98. doi: 10.1007/s12311-009-0111-y, PMID: 19452239

[60] Hoskovcová M Ulmanová O Šprdlík O Sieger T Novakova J Jech R. Disorders of balance and gait in essential tremor are associated with midline tremor and age. Cerebellum. (2012) 12:27–34. doi: 10.1007/s12311-012-0384-4, PMID: 22535593

[61] Nieuwboer A De Weerdt W Dom R Lesaffre E. A frequency and correlation analysis of motor deficits in Parkinson patients. Disabil Rehabil. (1998) 20:142–50. doi: 10.3109/09638289809166074 9571381

[62] Fuller R Van Winkle EP Anderson KE Gruber-Baldini AL Hill T Zampieri C. Dual task performance in Parkinson’s disease: a sensitive predictor of impairment and disability. Parkinsonism Relat Disord. (2013) 19:325–8. doi: 10.1016/j.parkreldis.2012.11.011, PMID: 23265679

[63] Juncos JL Lazarus J Graves-Allen E Shubeck L Rusin M Novak G. New clinical findings in the fragile X-associated tremor ataxia syndrome (FXTAS). Neurogenetics. (2011) 12:123–35. doi: 10.1007/s10048-010-0270-5, PMID: 21279400 PMC3766636

[64] O’Shea SD Morris ME Iansek R. Dual task interference during GAIT in people with Parkinson disease: effects of motor versus cognitive secondary tasks. Phys Ther. (2002) 82:888–97. doi: 10.1093/ptj/82.9.888, PMID: 12201803

[65] Rochester L Hetherington V Jones D Nieuwboer A Willems A Kwakkel G. Attending to the task: interference effects of functional tasks on walking in Parkinson’s disease and the roles of cognition, depression, fatigue, and balance. Arch Phys Med Rehabil. (2004) 85:1578–85. doi: 10.1016/j.apmr.2004.01.025 15468014

[66] Lin S Gao C Li H Huang P Ling Y Chen Z. Wearable sensor-based gait analysis to discriminate early Parkinson’s disease from essential tremor. J Neurol. (2023) 270:2283–301. doi: 10.1007/s00415-023-11577-6, PMID: 36725698 PMC10025195

[67] Robertson E Hall DA McAsey A O’Keefe JA. Fragile X-associated tremor/ataxia syndrome: phenotypic comparisons with other movement disorders. Clin Neuropsychol. (2016) 30:849–900. doi: 10.1080/13854046.2016.1202239, PMID: 27414076 PMC7336900

