# Supplementary material for: Digital gait markers to potentially distinguish fragile X-associated tremor/ataxia syndrome, Parkinson’s disease, and essential tremor
Source: Front Neurol. 2023 Dec 7;14:1308698. doi: 10.3389/fneur.2023.1308698 (PMC10755476; doi:10.3389/fneur.2023.1308698)
Supplement: Supplementary file 1 [file Data_Sheet_1.docx]

Supplementary Material

**
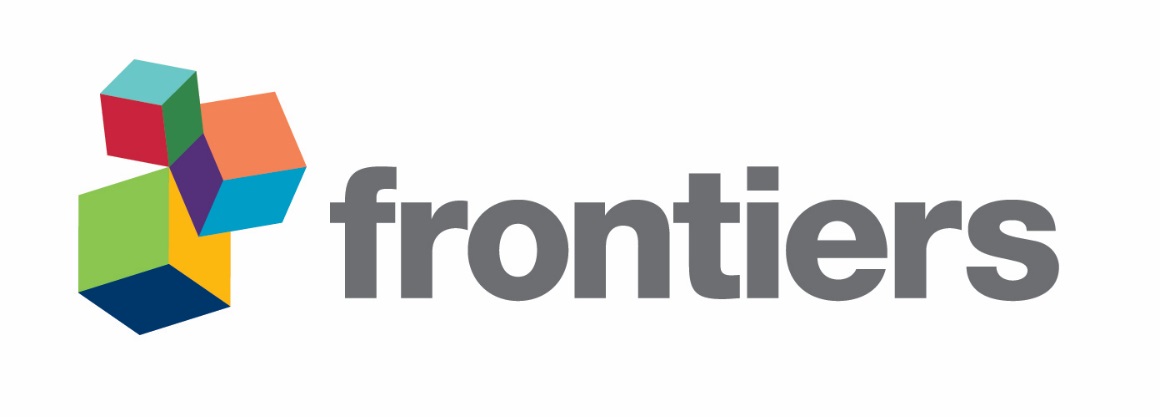
**

**Supplementary Table 1. Medications.** Summary of medications participants were taking for the treatment of movement disorder symptoms.

| **Medication** | **FXTAS**  **n (%)** | **PD**  **n (%)** | **ET**  **n (%)** |
| --- | --- | --- | --- |
| carbidopa-levodopa | 0 (0.00) | 17 (73.91) | 1 (5.00) |
| entacapone | 0 (0.00) | 3 (13.04) | 0 (0.00) |
| rasagiline | 0 (0.00) | 1 (4.35) | 0 (0.00) |
| pramipexole | 0 (0.00) | 2 (8.70) | 1 (5.00) |
| varenicline | 2 (9.52) | 1 (4.35) | 0 (0.00) |
| trihexyphenadyl | 0 (0.00) | 2 (8.70) | 0 (0.00) |
| propranolol | 7 (33.00) | 2 (8.70) | 12 (60.00) |
| selegiline | 0 (0.00) | 4 (17.39) | 0 (0.00) |
| benztropine | 0 (0.00) | 1 (4.35) | 0 (0.00) |
| amantadine | 3 (14.29) | 3 (13.04) | 0 (0.00) |
| rotigotine | 0 (0.00) | 1 (4.35) | 0 (0.00) |
| ropinorole | 0 (0.00) | 0 (0.00) | 1 (5.00) |
| gabapentin | 0 (0.00) | 0 (0.00) | 1 (5.00) |
| topamax | 1 (4.76) | 0 (0.00) | 0 (0.00) |
| Not on medication | 11 (52.38) | 3 (13.04) | 7 (35.00) |

**Supplementary Table 2. Correlations between modified FXTAS rating scale (FXTAS-RS) scores and self-selected (SS) speed two-minute walk test (2MWT) gait variables.** Spearman’s rho values with significant p-values (p ≤ 0.05) are shown in bold. * p ≤ 0.05, ** p ≤ 0.01

| **2MWT Gait Variables**  **Self-Selected (SS)** | **Controls**  **(n = 16)** | **FXTAS**  **(n = 16)** | **PD**  **(n = 14)** | **ET**  **(n = 10)** |
| --- | --- | --- | --- | --- |
|  | **FXTAS-RS** | | | |
| **Stride Length (%stature)** | -0.26 | **-0.57*** | -0.35 | -0.53 |
| **Stride Velocity (%stature/s)** | -0.24 | **-0.72**** | -0.32 | -0.21 |
| **Cadence (steps/min)** | -0.14 | -0.23 | -0.07 | 0.08 |
| **Double Limb Support (%)** | -0.09 | -0.20 | -0.26 | 0.18 |
| **Trunk Frontal ROM CoV** | 0.34 | -0.09 | 0.07 | -0.64 |
| **Stride Length (%stature) CoV** | 0.45 | 0.06 | 0.009 | -0.23 |
| **Stride Velocity (%stature/sec) CoV** | 0.38 | 0.20 | 0.07 | 0.02 |
| **Cadence (steps/min) CoV** | 0.34 | 0.21 | 0.02 | 0.02 |
| **Stride Length Asymmetry (%)** | 0.35 | 0.41 | -0.16 | -0.26 |
| **Arm Aymmetry Index (%)** | -0.01 | **0.58*** | -0.31 | 0.26 |
| **Turn Duration (sec)** | -0.30 | **0.72**** | 0.40 | 0.21 |
| **Number of Steps to Turn** | -0.50 | **0.57*** | 0.43 | 0.39 |
| **Peak Turn Velocity** | -0.12 | **-0.69**** | -0.21 | -0.33 |

**Supplementary Table 3. Correlations between modified FXTAS rating scale (FXTAS-RS) scores and fast as possible (FP) speed two-minute walk test (2MWT) gait variables.** Spearman’s rho values with significant p-values (p ≤ 0.05) are shown in bold. * p ≤ 0.05, ** p ≤ 0.01

| **2MWT Gait Variables**  **Fast as Possible (FP)** | **Controls**  **(n = 16)** | **FXTAS**  **(n = 16)** | **PD**  **(n = 14)** | **ET**  **(n = 10)** |
| --- | --- | --- | --- | --- |
|  | **FXTAS-RS** | | | |
| **Stride Length (%stature)** | -0.32 | -0.40 | -0.28 | -0.61 |
| **Stride Velocity (%stature/s)** | 0.04 | **-0.72**** | -0.33 | -0.43 |
| **Cadence (steps/min)** | 0.22 | **-0.54*** | 0.14 | 0.13 |
| **Double Limb Support (%)** | 0.08 | 0.06 | -0.15 | 0.06 |
| **Trunk Frontal ROM CoV** | 0.20 | -0.18 | 0.04 | -0.49 |
| **Stride Length (%stature) CoV** | 0.29 | -0.06 | -0.02 | -0.57 |
| **Stride Velocity (%stature/sec) CoV** | 0.15 | 0.04 | -0.12 | -0.32 |
| **Cadence (steps/min) CoV** | 0.19 | -0.04 | -0.11 | -0.23 |
| **Stride Length Asymmetry (%)** | 0.21 | 0.19 | -0.29 | -0.33 |
| **Arm Symmetry Index (%)** | -0.08 | 0.43 | -0.16 | 0.39 |
| **Turn Duration (sec)** | -0.13 | **0.72**** | 0.16 | 0.55 |
| **Number of Steps to Turn** | **-0.53*** | 0.42 | 0.36 | 0.55 |
| **Peak Turn Velocity** | -0.02 | **-0.63*** | -0.09 | -0.64 |

**Supplementary Table 4. Correlations between modified FXTAS rating scale (FXTAS-RS) scores and dual task (DT) two-minute walk test (2MWT) gait variables.** Spearman’s rho values with significant p-values (p ≤ 0.05) are shown in bold. * p ≤ 0.05, ** p ≤ 0.01

| **2MWT Gait Variables**  **Dual Task (DT)** | **Controls**  **(n = 16)** | **FXTAS**  **(n = 16)** | **PD**  **(n = 14)** | **ET**  **(n = 10)** |
| --- | --- | --- | --- | --- |
|  | **FXTAS-RS** | | | |
| **Stride Length (%stature)** | -0.21 | **-0.55*** | -0.18 | -0.54 |
| **Stride Velocity (%stature/s)** | -0.36 | **-0.74**** | -0.28 | -0.24 |
| **Cadence (steps/min)** | -0.45 | -0.31 | -0.08 | 0.06 |
| **Double Limb Support (%)** | 0.26 | -0.10 | -0.05 | 0.09 |
| **Trunk Frontal ROM CoV** | 0.25 | -0.24 | 0.13 | -0.51 |
| **Stride Length (%stature) CoV** | 0.08 | 0.32 | 0.12 | -0.32 |
| **Stride Velocity (%stature/sec) CoV** | 0.13 | 0.17 | 0.14 | -0.32 |
| **Cadence (steps/min) CoV** | 0.08 | 0.07 | 0.13 | -0.29 |
| **Stride Length Asymmetry (%)** | 0.17 | **0.55*** | -0.05 | -0.46 |
| **Arm Symmetry Index (%)** | 0.41 | 0.34 | -0.20 | -0.14 |
| **Turn Duration (sec)** | 0.27 | **0.52*** | 0.13 | 0.27 |
| **Number of Steps to Turn** | -0.24 | 0.50 | 0.30 | 0.09 |
| **Peak Turn Velocity** | -0.18 | **-0.61*** | -0.02 | -0.38 |

**Supplementary Table 5. Correlations between modified FXTAS rating scale (FXTAS-RS) scores and Instrumented Timed Up and Go test (i-TUG) gait variables.** Spearman’s rho values with significant p-values (p ≤ 0.05) are shown in bold. * p ≤ 0.05, ** p ≤ 0.01

| **i-TUG Gait Variables** | **Controls**  **(n = 16)** | **FXTAS**  **(n = 16)** | **PD**  **(n = 14)** | **ET**  **(n = 10)** |
| --- | --- | --- | --- | --- |
|  | **FXTAS-RS** | | | |
| **Total Duration** | 0.04 | -0.03 | **0.68**** | 0.46 |
| **Sit-to-Stand Duration (sec)** | -0.15 | 0.10 | 0.41 | 0.47 |
| **Sit-to-Stand Peak Velocity (deg/sec)** | -0.17 | 0.22 | -0.23 | -0.47 |
| **Turn-to-Sit Peak Turn Velocity (deg/sec)** | 0.12 | 0.09 | -0.41 | -0.61 |
| **Turn-to-Sit Duration (sec)** | 0.003 | -0.16 | **0.64*** | **0.73*** |
